# Depression and its Association With Cataract Status and Self-Perceived Burden of Elderly Patients With Cataract

**DOI:** 10.62641/aep.v53i5.2004

**Published:** 2025-10-05

**Authors:** Shanbo Zhou

**Affiliations:** ^1^Department of Ophthalmology, The Affiliated People’s Hospital of Ningbo University, 315040 Ningbo, Zhejiang, China

**Keywords:** cataract, depression, self-perceived burden, visual acuity

## Abstract

**Objective:**

This study aimed to explore the status of depressive mood in elderly patients with cataract and its association with visual acuity and self-perceived burden (SPB).

**Methods:**

A total of 210 senile patients with cataract attending the Affiliated People’s Hospital of Ningbo University between January 2025 and May 2025 were assessed with the scale, and 197 valid samples were finally obtained. Data on general demographics, underlying disease and best corrected visual acuity in both eyes were collected. The SPB scale (SPBS) and the self-rating depression scale (SDS) were used to assess the psychological state of the patients. Comparisons between groups were conducted using one-way analysis of variance and the relationship among visual acuity level, SPBS and SDS was analysed. Multiple logistic regression was performed for variables with *p* < 0.1 in univariate analysis to identify independent risk factors for depression.

**Results:**

Of the 197 patients, 84 were male and 113 were female, 59.39% were aged 65 years and older and 64.97% had visual acuity of less than 0.5. The SDS scores ranged from 34 to 70, with a mean of 49.60 ± 8.33, and the total SPBS scores ranged from 12 to 44, with a mean of 26.15 ± 7.69. The Pearson’s correlation showed that SDS was negatively correlated with visual acuity and significantly positively correlated with the total SPBS score and its dimensions. The results of multiple logistic regression suggested that age (OR = 1.051, *p* = 0.020), caregiver health status (OR = 1.968, *p* = 0.046) and diabetes mellitus (OR = 2.396, *p* = 0.038) were independent risk factors for depression in elderly patients with cataract.

**Conclusion:**

Elderly patients with cataract have a high prevalence of depression, which is significantly associated with SPB and depression. Visual acuity level, age, health status of caregivers and comorbid diabetes have a significant impact on the risk of depression.

## Introduction

The prevalence of various chronic degenerative diseases in the elderly population continues to increase as the world’s population ages, and cataract has become one of the most important blinding and vision-impairing diseases [1]. Recent global estimates suggest that cataract accounts for more than 39% of the causes of blindness worldwide [2]. Cataract usually presents as a progressive clouding of the lens, and early symptoms may be as simple as blurred vision or reduced night vision, which can eventually lead to severe visual impairment or even blindness if not treated in time. The prevalence of cataract in people aged 60 years and older increases considerably with age and is often comorbid with chronic diseases such as diabetes and hypertension, which increase the burden on public health and social care systems [2, 3]. Therefore, medical interventions and support for cataract urgently need to consider the needs of patients and society to achieve comprehensive health management.

The prevalence of depression and other adverse psychological conditions is significantly higher in elderly patients with cataract than in people without visual impairment due to reduced self-care, reduced social activities and weakened role functioning [4]. Patients with cataract who have low visual acuity have severe depressive mood and psychological distress, and this association is more pronounced in groups with advanced age and comorbid conditions and those without social support [4, 5]. Delayed surgical treatment, pessimistic expectations of postoperative outcomes and fear of decreased quality of life may be risk factors for depressive symptoms in patients with cataract [5, 6, 7]. Much research has focused on describing the association between cataract and depression, but the specific mechanisms of this association have been less explored. In particular, how vision loss may trigger depression by affecting patients’ psychological state and social support remains unclear.

The concept of self-perceived burden (SPB) has received increasing attention in recent years in many studies of elderly patients, especially in oncology research [8, 9]. It refers to negative emotions, such as guilt and shame, experienced by patients because of concerns that their illness or functional deficits will place an additional burden on their caregivers, family or society. Several surveys of elderly patients with chronic diseases and functional impairments have shown that SPB is positively correlated with depression level; increasing SPB not only adversely affects patients’ adherence to treatment but also weakens their social adjustment and sense of self-worth [10, 11]. Dry eye syndrome is associated with severe SPB and a high burden of psychological stress [12]. For elderly patients with cataract who require long-term care, gradually increasing visual impairment leads to further increase in their need for external help, which may lead to varying degrees of SPB; however, the relationship among cataract disease, SPB and depression has not been studied yet.

This study focused on elderly patients with cataract and examined the associations between visual acuity level and SPB and between depression and SPB. We quantified visual acuity to objectively assess the severity of cataract and combined it with SPB to determine their roles in depression in elderly patients with cataract. Results may provide a scientific basis for clinical staff to identify high-risk groups and develop multidimensional interventions. This work reveals new ideas for elucidating the causes of depression in the elderly and for establishing comprehensive prevention and treatment strategies.

## Methods

### Patients

This study included 210 elderly patients diagnosed with cataract at the Affiliated People’s Hospital of Ningbo University between January 2025 and May 2025. During cataract screening, the examiner used a direct ophthalmoscope to assess the red reflex from approximately 30 cm away from the patient’s pupil. Lens opacities were identified as localised dark areas against the uniform red reflex, indicating the presence of subtle lens changes. Large or dense opacities could lead to a diminished or absent red reflex. A slit-lamp biomicroscope was employed for detailed evaluation of the lens to determine the type (e.g., cortical, nuclear or posterior subcapsular), location and severity of opacification [13]. The inclusion criteria were as follows: diagnosis of cataract, age ≥60 years, basic communication skills, ability to cooperate in completing the questionnaire and voluntary participation. The exclusion criteria were combination of other serious eye diseases, presence of serious mental illness or cognitive impairment, malignant tumour and other serious organ damage. The study was approved by the Ethics Committee of the Affiliated People’s Hospital of Ningbo University (approval number: 2025-038), and all participants signed an informed consent form. The study was conducted in accordance with the principles of the Declaration of Helsinki.

### Data Collection

The following data were collected: demographic characteristics such as sex, age, education level, marital status, place of residence (urban and rural) and per capita household income; lifestyle habits such as smoking and drinking; number of chronic diseases such as dyslipidaemia, hypertension, diabetes and underlying diseases; and patient’s primary caregiver and their health status and type of health insurance billing. This study defined the condition of patients with cataract using best corrected visual acuity, which was measured by uniformly trained examiners with an international standard visual acuity chart under standard lighting conditions and recorded as a decimal visual acuity (0.1–1.0).

### Scales

Prior to the start of the study, investigators received uniform training to familiarise them with the content of the scale and the interview process. During the conduct of the study, patients were assessed using the scale in an outpatient or inpatient setting. For respondents with limited vision or literacy, investigators read aloud and recorded patient’s responses item by item to ensure concise language and accurate explanations when reading the questions to minimise information bias.

The SPB Scale (SPBS, Cronbach’s alpha = 0.91) was used to assess negative emotions, such as guilt and remorse, felt by patients when they may be burdened by the disease to their caregivers and families. The scale consists of 10 items (two items in the physical burden dimension, six items in the emotional burden dimension and two items in the economic burden dimension) and is scored on a 5-point Likert scale. The total score is 50, and higher scores indicate greater SPB. A total score of <20 indicates no burden, a score of 20–29 indicates mild burden, a score of 30–39 indicates moderate burden and a score of ≥40 indicates severe burden [14, 15].

We assessed patients’ depression by using the Self-Rating Depression Scale (SDS, Cronbach’s alpha = 0.78), which consists of 20 items, each rated on a scale of 1–4. The total score is 80, and a standardised score was obtained by multiplying the scale score by 1.25. A score of 50–59 (standardised) is usually considered mild depression, 60–69 indicates moderate depression and ≥70 indicates severe depression [16, 17].

### Statistical Analysis

Data were cross-checked by two researchers using Excel software (version 2021, Microsoft, Redmond, WA, USA) and analysed using Statistical Product and Service Solutions 26.0 (IBM, Armonk, NY, USA). All measurement data were tested for normality by using the Shapiro–Wilk test. The data had normal distribution and were therefore expressed as mean ± standard deviation (SD). Categorical data were presented as number of cases (*n*) and percentage (%). For continuous variables, independent samples *t*-tests were used for two-group comparisons. For categorical variables, Chi-squared tests or Fisher’s exact tests were used as appropriate. Pearson correlation analysis was used to evaluate the relationship among visual acuity level, SPB and depression by determining their correlation coefficients. Multiple logistic regression was used to analyse risk factors for the development of depression in patients with cataract. No significant multicollinearity was detected among the independent variables, as all variance inflation factors were below 3.0. *p* < 0.05 indicates a statistically significant difference.

## Results

### Patient Characteristics

A total of 197 white elderly patients with cataract had complete information and successfully completed the scale. As shown in Table 1, 42.64% of the patients were male, 59.39% were in the age group of >65 years and 64.97% had visual acuity <0.5. Most of the patients had a low level of education, lived in urban areas and had a per capita household income of 3000–5000 RMB (1 RMB = 0.1392 USD). In terms of chronic diseases, 55.84% of the patients had diabetes mellitus, 65.48% had hypertension and 45.18% had dyslipidaemia. Moreover, 38.07% had 2 chronic diseases and 2.54% had 4 or more chronic diseases.

**Table 1.** **Patient characteristics**.

Variables		*n* (*N* = 197)	%
Sex			
	Male	84	42.64
	Female	113	57.36
Age (years)			
	≤65	80	40.61
	>65	117	59.39
Visual acuity			
	<0.5	128	64.97
	≥0.5	69	35.03
Educational level			
	Primary school or below	122	61.93
	Junior high school	62	31.47
	Senior high school or above	13	6.60
Marital status			
	Married	126	63.96
	Widowed	65	32.99
	Unmarried (never married, divorced or separated)	6	3.05
Residence			
	Rural	71	36.04
	Urban	126	63.96
Per capita household income (RMB, 1 RMB = 0.1392 USD)			
	<3000	44	22.34
	3000–5000	105	53.30
	>5000	48	24.37
Family caregiver			
	Spouse	53	26.90
	Children	108	54.82
	Other	36	18.27
Health status of the caregiver			
	Healthy	133	67.51
	Unhealthy	64	32.49
Type of reimbursement method			
	Resident insurance	75	38.07
	Employee insurance	122	61.93
Smoking			
	No	98	49.75
	Yes	99	50.25
Alcohol consumption			
	No	114	57.87
	Yes	83	42.13
Diabetes			
	No	87	44.16
	Yes	110	55.84
Hypertension			
	No	68	34.52
	Yes	129	65.48
Dyslipidaemia			
	No	108	54.82
	Yes	89	45.18
History of stroke			
	No	161	81.73
	Yes	36	18.27
Number of underlying diseases			
	0	15	7.61
	1	56	28.43
	2	75	38.07
	3	46	23.35
	≥4	5	2.54

### SDS and SPBS Scores

Table 2 shows that the range of SDS scores was 34–70, with a mean value of 49.60 ± 8.33, and that of the total SPBS scores was 12–44, with a mean value of 26.15 ± 7.69. The scores for the three dimensions of SPBS were as follows: emotional burden, 15.86 ± 5.93 (range 6–27); physical burden, 5.42 ± 2.12 (range 2–10); and economic burden, 4.86 ± 1.92 (range 2–9).

**Table 2.** **Self-perceived burden scale and self-rating depression scale scores of the patients**.

Variables		Range (min–max)	mean ± SD
SDS		34–70	49.60 ± 8.33
SPBS		12–44	26.15 ± 7.69
	Physical burden	2–10	5.42 ± 2.12
	Emotional burden	6–27	15.86 ± 5.93
	Economic burden	2–9	4.86 ± 1.92

SPBS, Self-Perceived Burden Scale; SDS, Self-Rating Depression Scale.

### SDS and SPBS Classification of Patients

Based on the classification criteria of SDS and SPBS (Table 3), 55.33% of the patients had SDS within the normal range, 29.95% were mildly depressed, 13.71% were moderately depressed and 1.02% were severely depressed. In terms of SPBS, 33.50% of the patients had no significant burden, 36.04% had mild burden, 26.90% had moderate burden and 3.55% had severe burden.

**Table 3.** **Distribution and mean scores of self-rating depression scale and self-perceived burden scale by severity levels**.

Variables		*n* (*N* = 197)	%	mean ± SD
SDS				
	Normal	109	55.33	43.32 ± 3.97
	Mild depression	59	29.95	54.58 ± 3.17
	Moderate depression	27	13.71	62.59 ± 2.42
	Severe depression	2	1.02	70.00 ± 0.00
SPBS				
	No burden	66	33.50	17.91 ± 1.48
	Mild burden	71	36.04	25.45 ± 2.70
	Moderate burden	53	26.90	35.36 ± 2.57
	Severe burden	7	3.55	41.14 ± 1.35

SPBS, Self-Perceived Burden Scale; SDS, Self-Rating Depression Scale. SDS: <50, no depression; 50–59, mild depression; 60–69, moderate depression; and ≥70, severe depression. SPBS: <20, no burden; 20–29, mild burden; 30–39, moderate burden; and ≥40, severe burden.

### Univariate Analysis of Depressed and Non-Depressed Groups

The depressed and non-depressed groups were defined according to the criteria with SDS scores ≥50 as the cutoff (Table 4). Age (*p* = 0.020), visual acuity level (*p* = 0.033) and caregiver health status (*p* = 0.050) were significantly different between the two groups. Factors such as diabetes mellitus (*p* = 0.091) and history of stroke (*p* = 0.068) had slightly larger *p*-values but remained less than 0.1. In this regard, their potential effects on depression need to be considered in subsequent multivariate analyses.

**Table 4.** **Univariate analysis between depression and non-depression groups**.

Variables		Non-depression group (*n* = 109)	Depression group (*n* = 88)	t/χ^2^	*p* value
Sex, *n* (%)				0.52	0.473
	Male	44 (40.37)	40 (45.45)		
	Female	65 (59.63)	48 (54.55)		
Age, mean ± SD		68.45 ± 7.05	71.06 ± 8.57	–2.34	0.020
Visual acuity, mean ± SD		0.42 ± 0.11	0.38 ± 0.13	2.14	0.033
Educational level, *n* (%)				0.03	0.987
	Primary school or below	68 (62.39)	54 (61.36)		
	Junior high school	34 (31.19)	28 (31.82)		
	Senior high school or above	7 (6.42)	6 (6.82)		
Marital status, *n* (%)				-	0.254
	Married	75 (68.81)	51 (57.95)		
	Widowed	31 (28.44)	34 (38.64)		
	Unmarried (never married, divorced or separated)	3 (2.75)	3 (3.41)		
Residence, *n* (%)				0.96	0.327
	Rural	36 (33.03)	35 (39.77)		
	Urban	73 (66.97)	53 (60.23)		
Per capita household income (RMB, 1 RMB = 0.1392 USD), *n* (%)				2.20	0.333
	<3000	23 (21.10)	21 (23.86)		
	3000–5000	55 (50.46)	50 (56.82)		
	>5000	31 (28.44)	17 (19.32)		
Family caregiver, *n* (%)				0.28	0.869
	Spouse	30 (27.52)	23 (26.14)		
	Children	58 (53.21)	50 (56.82)		
	Other	21 (19.27)	15 (17.05)		
Health status of the caregiver, *n* (%)				3.85	0.050
	Healthy	80 (73.39)	53 (60.23)		
	Unhealthy	29 (26.61)	35 (39.77)		
Type of reimbursement method, *n* (%)				2.64	0.104
	Resident insurance	47 (43.12)	28 (31.82)		
	Employee insurance	62 (56.88)	60 (68.18)		
Smoking, *n* (%)				1.87	0.171
	No	59 (54.13)	39 (44.32)		
	Yes	50 (45.87)	49 (55.68)		
Alcohol consumption, *n* (%)				0.10	0.755
	No	62 (56.88)	52 (59.09)		
	Yes	47 (43.12)	36 (40.91)		
Diabetes, *n* (%)				2.86	0.091
	No	54 (49.54)	33 (37.50)		
	Yes	55 (50.46)	55 (62.50)		
Hypertension, *n* (%)				0.17	0.678
	No	39 (35.78)	29 (32.95)		
	Yes	70 (64.22)	59 (67.05)		
Dyslipidaemia, *n* (%)				0.05	0.828
	No	59 (54.13)	49 (55.68)		
	Yes	50 (45.87)	39 (44.32)		
History of stroke, *n* (%)				3.33	0.068
	No	94 (86.24)	67 (76.14)		
	Yes	15 (13.76)	21 (23.86)		
Number of underlying diseases, *n* (%)				7.26	0.123
	0	13 (11.93)	2 (2.27)		
	1	29 (26.61)	27 (30.68)		
	2	42 (38.53)	33 (37.50)		
	3	23 (21.10)	23 (26.14)		
	≥4	2 (1.83)	3 (3.41)		
SPBS		23.84 ± 6.44	29.00 ± 8.17	–4.95	<0.001

SPBS, Self-Perceived Burden Scale.

### Univariate Analysis Between Burden and Non-Burden Groups

As shown in Table 5, a total SPBS score ≥20 was defined as the burden group (mild and above). Age (*p* = 0.032), diabetes (*p* = 0.017) and dyslipidaemia (*p* = 0.029) were significantly different between the burden and non-burden groups. The other indicators had no significant difference between the two groups.

**Table 5.** **Univariate analysis between burden and non-burden groups**.

Variables		Non-burden group	Burden group	t/χ^2^	*p* value
Sex, *n* (%)				0.43	0.513
	Male	26 (39.39)	58 (44.27)		
	Female	40 (60.61)	73 (55.73)		
Age, mean ± SD		68.08 ± 6.30	70.39 ± 8.45	–2.16	0.032
Visual acuity, mean ± SD		0.41 ± 0.13	0.39 ± 0.11	1.06	0.289
Educational level, *n* (%)				1.45	0.484
	Primary school or below	37 (56.06)	85 (64.89)		
	Junior high school	24 (36.36)	38 (29.01)		
	Senior high school or above	5 (7.58)	8 (6.11)		
Marital status, *n* (%)				-	0.680
	Married	42 (63.64)	84 (64.12)		
	Widowed	21 (31.82)	44 (33.59)		
	Unmarried (never married, divorced or separated)	3 (4.55)	3 (2.29)		
Residence, *n* (%)				0.48	0.487
	Rural	26 (39.39)	45 (34.35)		
	Urban	40 (60.61)	86 (65.65)		
Per capita household income (RMB, 1 RMB = 0.1392 USD), *n* (%)				0.14	0.934
	<3000	14 (21.21)	30 (22.90)		
	3000–5000	35 (53.03)	70 (53.44)		
	>5000	17 (25.76)	31 (23.66)		
Family caregiver, *n* (%)				2.39	0.302
	Spouse	16 (24.24)	37 (28.24)		
	Children	34 (51.52)	74 (56.49)		
	Other	16 (24.24)	20 (15.27)		
Health status of the caregiver, *n* (%)				2.05	0.152
	Healthy	49 (74.24)	84 (64.12)		
	Unhealthy	17 (25.76)	47 (35.88)		
Type of reimbursement method, *n* (%)				2.54	0.111
	Resident insurance	20 (30.30)	55 (41.98)		
	Employee insurance	46 (69.70)	76 (58.02)		
Smoking, *n* (%)				0.31	0.580
	No	31 (46.97)	67 (51.15)		
	Yes	35 (53.03)	64 (48.85)		
Alcohol consumption, *n* (%)				0.06	0.805
	No	39 (59.09)	75 (57.25)		
	Yes	27 (40.91)	56 (42.75)		
Diabetes, *n* (%)				5.70	0.017
	No	37 (56.06)	50 (38.17)		
	Yes	29 (43.94)	81 (61.83)		
Hypertension, *n* (%)				0.06	0.804
	No	22 (33.33)	46 (35.11)		
	Yes	44 (66.67)	85 (64.89)		
Dyslipidaemia, *n* (%)				4.75	0.029
	No	29 (43.94)	79 (60.31)		
	Yes	37 (56.06)	52 (39.69)		
History of stroke, *n* (%)				1.43	0.232
	No	57 (86.36)	104 (79.39)		
	Yes	9 (13.64)	27 (20.61)		
Number of underlying diseases, *n* (%)				2.93	0.569
	0	7 (10.61)	8 (6.11)		
	1	16 (24.24)	40 (30.53)		
	2	28 (42.42)	47 (35.88)		
	3	13 (19.70)	33 (25.19)		
	≥4	2 (3.03)	3 (2.29)		
SDS		45.56 ± 6.10	51.64 ± 8.57	–5.14	<0.001

SDS, Self-Rating Depression Scale.

### Correlation Analysis of SDS With Visual Acuity and SPBS

**Fig. 1.**
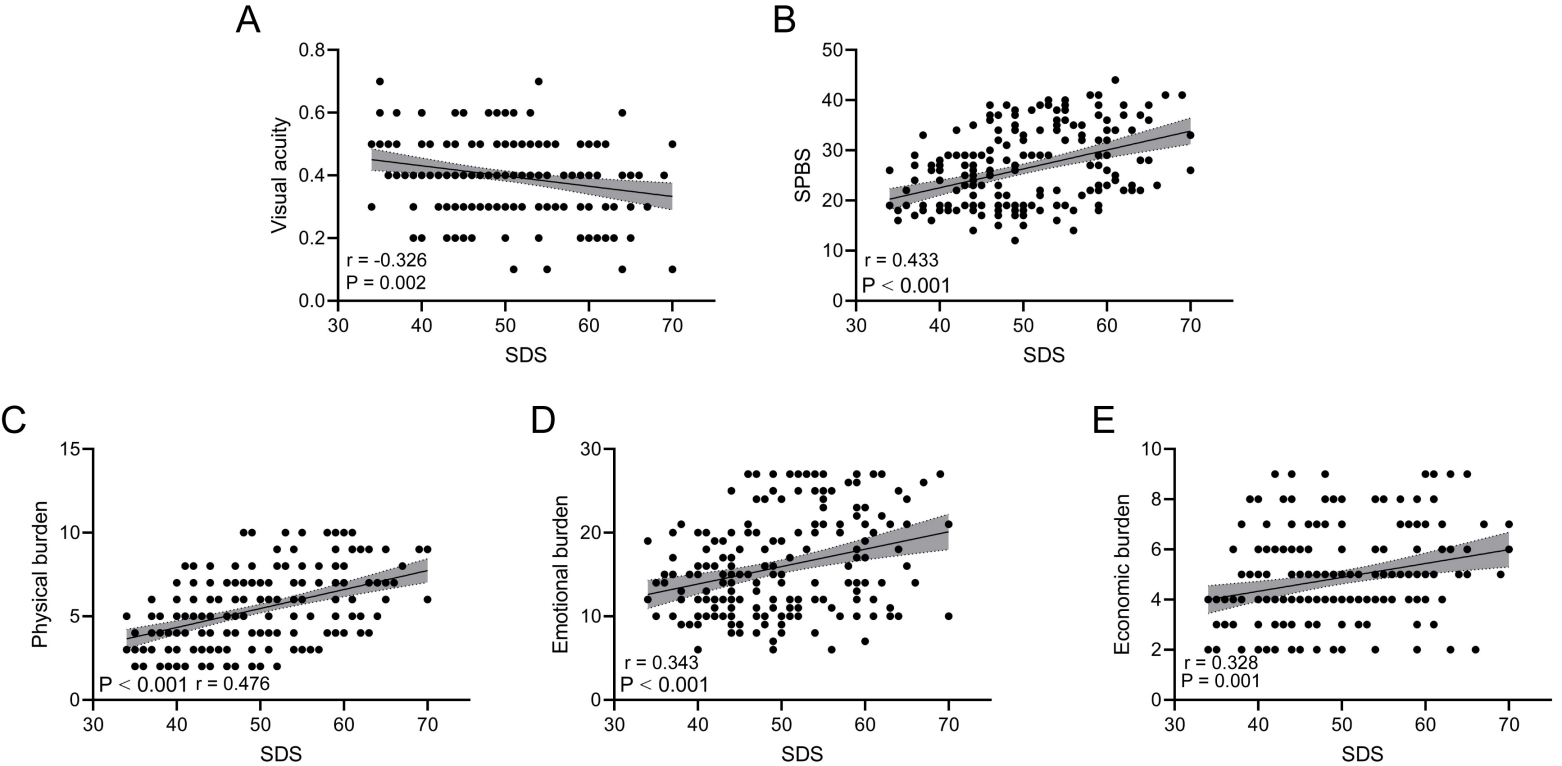
**Correlation analysis of self-rating depression scale (SDS) with visual acuity and self-perceived burden scale (SPBS)**. (A) Scatterplot of correlation between visual acuity and SDS. (B) Scatterplot of correlation between SPBS score and SDS. (C) Scatterplot of correlation between physical burden score and SDS. (D) Scatterplot of correlation between emotional burden score and SDS. (E) Scatterplot of correlation between economic burden score and SDS.

Fig. 1 shows the scatter distribution and regression trends between SDS scores and visual acuity, total SPBS score and its three dimensions. The results showed that SDS was negatively correlated with visual acuity (Fig. 1A, *p* = 0.002, r = –0.326) and significantly positively correlated with the total SPBS score (Fig. 1B, *p* < 0.001, r = 0.433) as well as the physical burden (Fig. 1C, *p* < 0.001, r = 0.476), emotional burden (Fig. 1D, *p* < 0.001, r = 0.343) and economic burden (Fig. 1E, *p* = 0.001, r = 0.328) of SPBS. This finding indicates that patients with higher levels of depression tended to have lower levels of visual acuity and higher SPB (including all dimensions). All reported *p* values and correlation coefficients were obtained from analyses adjusted for potential confounders, including age and educational level.

### Multiple Logistic Regression Analysis of Depression

Variables with *p* < 0.1 in Table 4 (age, visual acuity, caregiver health status, diabetes mellitus and history of stroke) were included in multiple logistic regression. These variables were coded as follows: caregiver health status (0 = healthy, 1 = unhealthy), diabetes mellitus (0 = no, 1 = yes) and history of stroke (0 = no, 1 = yes). Age and visual acuity were treated as continuous variables. As shown in Table 6, the factors with independent effects on depression included age (OR = 1.051, *p* = 0.020), caregiver health status (OR = 1.968, *p* = 0.046) and diabetes (OR = 2.396, *p* = 0.038). These results suggest that older patients with cataract and poor caregiver health and comorbid diabetes are more likely to be depressed.

**Table 6.** **Multiple logistic regression analysis of depression in patients with cataract**.

Variables		β	S.E	Wald	*p* value	Odds ratio (95% CI)
Age		0.049	0.021	5.432	0.020	1.051 (1.008–1.095)
Health status of the caregiver						
	Healthy					Reference
	Unhealthy	0.677	0.340	3.964	0.046	1.968 (1.011–3.833)
Diabetes						
	No					Reference
	Yes	0.874	0.420	4.326	0.038	2.396 (1.052–5.459)

## Discussion

This study focused on elderly patients with cataract and analysed three aspects of visual acuity level, depressive status and SPB. The results revealed a high percentage of comorbid depression and a prevalence of SPB. Moreover, SPB was significantly and positively correlated with depression. Multifactorial analysis further identified age, caregiver health status and diabetes as independent risk factors for depression.

The SDS scores of more than 40% of the patients reached the threshold for depression, which is consistent with previous findings that elderly patients with cataract are vulnerable to psychological problems [2, 4, 5]. As lens clouding progresses, patients often experience a decline in visual acuity and a limitation of daily activities. The patients are also susceptible to anxiety, loneliness and reduced self-efficacy. Moreover, persistent visual impairment can exacerbate depression if patients do not undergo surgery or effective intervention in a timely manner [7, 18]. Thus far, the relationship between visual acuity levels and depression in patients with cataract remains a subject of debate. Early studies indicated that patients with poor preoperative visual acuity exhibited a nearly 60% elevated risk of depression compared with patients with better visual acuity [5, 19]. However, another study found no significant association between the severity of visual impairment and the risk of depression in patients with cataract [20]. The present study reported a significant negative correlation between visual acuity level and depression and that patients in the depressed group had significantly lower visual acuity than patients in the non-depressed group, suggesting a strong correlation between visual acuity status and mood disorders. The inconsistent findings regarding the association between visual acuity and depression may stem from multiple underlying mechanisms. First, visual impairment can directly limit daily activities and social participation, leading to social isolation and reduced self-efficacy, which are known risk factors for depression [21]. Second, the neural pathway hypothesis suggests that cataract and depression may share common neurobiological substrates, such as dysregulation of the hypothalamic–pituitary–adrenal axis or chronic inflammation [22]. Third, the psychological impact likely follows a threshold effect, where depression risk increases significantly only when visual acuity deteriorates beyond a certain functional level (e.g., difficulty in recognising faces). This phenomenon could explain why some studies with predominantly mild impairment cases failed to detect significant associations [20].

In this study, the SPBS was utilised to evaluate the psychological burden of guilt, guilt and worry that patients may experience due to their condition. Approximately two-thirds of the patients exhibited varying degrees of SPB (≥20 points), and that the total SPBS scores and the dimensions (physical, emotional, and, economic) were significantly associated with depression. The concept of SPB was initially proposed in studies of cancer and other major or life-threatening diseases and has gradually expanded to the field of chronic diseases and dysfunction [8, 23, 24]. Patients with ocular diseases who frequently require assistance from family or society during the course of their illnesses are more likely to have negative self-perceptions regarding finances, caregiving and emotions. These adverse emotions have been identified as significant triggers for depression [12, 25]. The present findings are consistent with these perspectives, suggesting that SPB can be a significant dimension in the psychological assessment of elderly patients with cataract.

Multiple logistic regression showed that older patients with advanced age had a significantly higher risk of depression, which may be related to factors such as chronic diseases that increase with age and diminished social roles. Previous studies also found a significantly higher risk of anxiety and depression in older patients with cataract [6, 26, 27]. Poor caregiver health also emerged as an independent risk factor for depression. When family or primary caregivers are in poor health themselves, patients may have more difficulty accessing external support and may worry about their caregiver’s health, thereby increasing psychological distress and negative emotions [28]. Interestingly, the present study did not find a significant effect of vision level on the final multiple regression model possibly because the sample size was not sufficiently large to highlight its independent contribution. Diabetes mellitus was also included as an independent risk factor possibly because it is a disease that predisposes patients to depressive symptoms; moreover, poor long-term glycaemic control and complications such as retinopathy resulting from this disease may further reduce ocular conditions and quality of life in older adults, making them more susceptible to or have exacerbating depressive moods [29, 30, 31].

Routine ocular assessment and surgical planning for elderly patients with cataract should be accompanied by evaluation of psychosocial status and social support needs. Based on our findings, we propose strategies to mitigate SPB in elderly patients with cataract. Firstly, psychosocial support interventions should focus on reducing patients’ perceived burden through caregiver training. Educating family members on effective communication and caregiving skills can alleviate patients’ guilt about relying on others. For example, structured programs that emphasise shared decision-making in treatment plans may enhance patients’ sense of autonomy. Secondly, vision rehabilitation services could address the root cause of SPB by improving functional vision. Early referral to low-vision clinics for assistive devices (e.g., magnifiers, electronic aids) may reduce dependence on caregivers, thereby decreasing physical and emotional burden [3]. Surgical intervention for cataract should also be prioritised to restore vision and mitigate long-term SPB. Thirdly, multidisciplinary mental health support is warranted, particularly for patients with comorbid diabetes or older age. Psychologists should be integrated into ophthalmology care teams to provide cognitive–behavioural therapy to help reframe negative self-perception.

This study has some limitations. Firstly, it is a single-centre study, so multi-centre studies or interventional randomised controlled trials are necessary to verify causal inference. Some of the scale (e.g., SDS, SPBS) are self-assessment tools. Future studies should consider the introduction of clinical diagnostic scales or objective examination indicators. They should also expand the sample size and conduct follow up to further explore the dynamic evolution of the depression status of elderly patients with cataract. Furthermore, given the substantial impact of caregivers’ health status on patients’ psychology, caregivers’ psychological characteristics should be assessed to investigate the interplay between family environment, social support, accessibility of medical resources and other factors. This endeavour will provide a comprehensive foundation for management of the elderly population with cataract. Finally, the present study used only ‘best-corrected visual acuity’ for visual acuity assessment and did not distinguish between monocular and binocular visual impairment. This method of assessment may simplify the impact of vision loss to some extent because unilateral and bilateral visual impairments may have different effects on patients’ daily functioning and psychological distress. Future studies should differentiate between monocular and binocular visual impairment to fully assess the impact of vision loss on patients’ psychological status.

## Conclusion

Elderly patients with cataract have a high prevalence of depression, and a significant association exists between SPB and depression. Visual acuity level, age, health status of caregivers and comorbid diabetes have a significant impact on the risk of depression.

## Availability of Data and Materials

All experimental data included in this study can be obtained by contacting the first author if needed.

## References

[1] Hashemi H, Pakzad R, Yekta A, Aghamirsalim M, Pakbin M, Ramin S (2020). Global and regional prevalence of age-related cataract: a comprehensive systematic review and meta-analysis. *Eye (London, England)*.

[2] Vision Loss Expert Group of the Global Burden of Disease Study, GBD 2019 Blindness and Vision Impairment Collaborators (2024). Global estimates on the number of people blind or visually impaired by cataract: a meta-analysis from 2000 to 2020. *Eye (London, England)*.

[3] Ang MJ, Afshari NA (2021). Cataract and systemic disease: A review. *Clinical & Experimental Ophthalmology*.

[4] Chen PW, Liu PPS, Lin SM, Wang JH, Huang HK, Loh CH (2020). Cataract and the increased risk of depression in general population: a 16-year nationwide population-based longitudinal study. *Scientific Reports*.

[5] Freeman EE, Gresset J, Djafari F, Aubin MJ, Couture S, Bruen R (2009). Cataract-related vision loss and depression in a cohort of patients awaiting cataract surgery. *Canadian Journal of Ophthalmology. Journal Canadien D’ophtalmologie*.

[6] Wang T, Li H, Cao Q (2024). Age-related cataract without surgery is related to exacerbated depression symptoms: a cross-sectional study of Chinese adults from Anhui, China. *Frontiers in Medicine*.

[7] Wang S, Du Z, Lai C, Seth I, Wang Y, Huang Y (2024). The association between cataract surgery and mental health in older adults: a review. *International Journal of Surgery (London, England)*.

[8] Saji A, Oishi A, Harding R (2023). Self-perceived Burden for People With Life-threatening Illness: A Qualitative Systematic Review. *Journal of Pain and Symptom Management*.

[9] Li K, Zhu L, Zhang LY (2023). Correlations between activation, family adaptation, and self-perceived burden in breast cancer patients with an implanted venous access port: A cross-sectional study. *Medicine*.

[10] Salazar RD, Weizenbaum E, Ellis TD, Earhart GM, Ford MP, Dibble LE (2019). Predictors of self-perceived stigma in Parkinson’s disease. *Parkinsonism & Related Disorders*.

[11] Dempsey LE, Karver MS, Labouliere C, Zesiewicz TA, De Nadai AS (2012). Self-perceived burden as a mediator of depression symptoms amongst individuals living with a movement disorder. *Journal of Clinical Psychology*.

[12] Wang MT, Muntz A, Wolffsohn JS, Craig JP (2021). Association between dry eye disease, self-perceived health status, and self-reported psychological stress burden. *Clinical & Experimental Optometry*.

[13] Miller KM, Oetting TA, Tweeten JP, Carter K, Lee BS, Lin S (2022). Cataract in the Adult Eye Preferred Practice Pattern. *Ophthalmology*.

[14] Cousineau N, McDowell I, Hotz S, Hébert P (2003). Measuring chronic patients’ feelings of being a burden to their caregivers: development and preliminary validation of a scale. *Medical Care*.

[15] Zhu Y, Xu H, Ding D, Liu Y, Guo L, Zauszniewski JA (2023). Resourcefulness as a mediator in the relationship between self-perceived burden and depression among the young and middle-aged stroke patients: A cross-sectional study. *Heliyon*.

[16] ZUNG WW (1965). A SELF-RATING DEPRESSION SCALE. *Archives of General Psychiatry*.

[17] Tian Y, Wang Y, Li J, Wang M, Dang S (2019). Evaluation of reliability and validity of self-rating anxiety scale and self-rating depression scale in patients with liver cirrhosis. *Journal of Practical Hepatology*.

[18] Xiang Q, Li F, Wang H, ZeLang L, Liu Q, Luo Y (2022). Correlation analysis between self-rated treatment effect and diagnosis and treatment of elderly poor cataract patientswith poor financial condition in rural areas of Ganzi Prefecture. *American Journal of Translational Research*.

[19] Choi HG, Lee MJ, Lee SM (2018). Visual impairment and risk of depression: A longitudinal follow-up study using a national sample cohort. *Scientific Reports*.

[20] Parravano M, Petri D, Maurutto E, Lucenteforte E, Menchini F, Lanzetta P (2021). Association Between Visual Impairment and Depression in Patients Attending Eye Clinics: A Meta-analysis. *JAMA Ophthalmology*.

[21] Patrick RE, Dickinson RA, Gentry MT, Kim JU, Oberlin LE, Park S (2024). Treatment resistant late-life depression: A narrative review of psychosocial risk factors, non-pharmacological interventions, and the role of clinical phenotyping. *Journal of Affective Disorders*.

[22] Cui L, Li S, Wang S, Wu X, Liu Y, Yu W (2024). Major depressive disorder: hypothesis, mechanism, prevention and treatment. *Signal Transduction and Targeted Therapy*.

[23] Tan M, Liu Y, Zhao R, Li H (2023). The effect of pain social support on kinesiophobia in older patients with rheumatoid arthritis: The mediating role of self-perceived burden. *Geriatric Nursing (New York, N.Y.)*.

[24] Zhang Y, Li X, Bi Y, Kan Y, Liu H, Liu L (2023). Effects of family function, depression, and self-perceived burden on loneliness in patients with type 2 diabetes mellitus: a serial multiple mediation model. *BMC Psychiatry*.

[25] Havstam Johansson L, Zetterberg M, Falk Erhag H (2025). Self-perceived and measured visual function, the impact of eye-disease, wellbeing, social determinants, and personality traits in Swedish 70-year-olds-results from the Gothenburg H70 Study. *Acta Ophthalmologica*.

[26] Hwang G, Lee SH, Lee DY, Park C, Roh HW, Son SJ (2025). Age-related eye diseases and subsequent risk of mental disorders in older adults: A real-world multicenter study. *Journal of Affective Disorders*.

[27] Brunes A, Heir T (2020). Visual impairment and depression: Age-specific prevalence, associations with vision loss, and relation to life satisfaction. *World Journal of Psychiatry*.

[28] Yu Q, Xu L, Zhou L, Jiang X, Xu N, Lang Z (2025). A study of the relationship between social support, caregiving experience and depressed mood of family caregivers of home-dwelling older adults with disability- - a cross-sectional study. *BMC Public Health*.

[29] Teo ZL, Tham YC, Yu M, Chee ML, Rim TH, Cheung N (2021). Global Prevalence of Diabetic Retinopathy and Projection of Burden through 2045: Systematic Review and Meta-analysis. *Ophthalmology*.

[30] Liu Y, Huang SY, Liu DL, Zeng XX, Pan XR, Peng J (2024). Bidirectional relationship between diabetes mellitus and depression: Mechanisms and epidemiology. *World Journal of Psychiatry*.

[31] Qiu H (2024). Prevalence and risk factors of anxiety and depression in diabetic retinopathy patients: A cross-sectional study using multiple scales. *European Journal of Ophthalmology*.

